# Genomic landscape and actionable mutations of brain metastases derived from non–small cell lung cancer: A systematic review

**DOI:** 10.1093/noajnl/vdad145

**Published:** 2023-11-24

**Authors:** Lily J Andrews, Zak A Thornton, Ruqiya Saleh, Sarah Dawson, Susan C Short, Richard Daly, Julian P T Higgins, Philippa Davies, Kathreena M Kurian

**Affiliations:** MRC Integrative Epidemiology Unit (IEU), Bristol Medical School, University of Bristol, Bristol, UK; Population Health Sciences, Bristol Medical School, University of Bristol, Bristol, UK; Cancer Research Integrative Cancer Epidemiology Programme, University of Bristol, Bristol, UK; MRC Integrative Epidemiology Unit (IEU), Bristol Medical School, University of Bristol, Bristol, UK; Population Health Sciences, Bristol Medical School, University of Bristol, Bristol, UK; Cancer Research Integrative Cancer Epidemiology Programme, University of Bristol, Bristol, UK; Bristol Medical School, University of Bristol, Bristol, UK; Population Health Sciences, Bristol Medical School, University of Bristol, Bristol, UK; Leeds Institute of Medical Research, University of Leeds, Leeds, UK; Cellular Pathology Department, North Bristol NHS Trust, Bristol, UK; Population Health Sciences, Bristol Medical School, University of Bristol, Bristol, UK; MRC Integrative Epidemiology Unit (IEU), Bristol Medical School, University of Bristol, Bristol, UK; Population Health Sciences, Bristol Medical School, University of Bristol, Bristol, UK; Cancer Research Integrative Cancer Epidemiology Programme, University of Bristol, Bristol, UK; MRC Integrative Epidemiology Unit (IEU), Bristol Medical School, University of Bristol, Bristol, UK; Population Health Sciences, Bristol Medical School, University of Bristol, Bristol, UK; Cancer Research Integrative Cancer Epidemiology Programme, University of Bristol, Bristol, UK; Brain Tumour Research Centre, Bristol Medical School, University of Bristol, Bristol, UK

**Keywords:** brain metastases, non–small cell lung cancer, genomics, actionable mutations

## Abstract

**Background:**

Brain metastases derived from non–small cell lung cancer (NSCLC) represent a significant clinical problem. We aim to characterize the genomic landscape of brain metastases derived from NSCLC and assess clinical actionability.

**Methods:**

We searched Embase, MEDLINE, Web of Science, and BIOSIS from inception to 18/19 May 2022. We extracted information on patient demographics, smoking status, genomic data, matched primary NSCLC, and programmed cell death ligand 1 expression.

**Results:**

We found 72 included papers and data on 2346 patients. The most frequently mutated genes from our data were *EGFR* (*n* = 559), *TP53* (*n* = 331), *KRAS* (*n* = 328), *CDKN2A* (*n* = 97), and *STK11* (*n* = 72). Common missense mutations included *EGFR* L858R (*n* = 80) and *KRAS* G12C (*n* = 17). Brain metastases of ever versus never smokers had differing missense mutations in *TP53* and *EGFR*, except for L858R and T790M in *EGFR*, which were seen in both subgroups. Of the top 10 frequently mutated genes that had primary NSCLC data, we found 37% of the specific mutations assessed to be discordant between the primary NSCLC and brain metastases.

**Conclusions:**

To our knowledge, this is the first systematic review to describe the genomic landscape of brain metastases derived from NSCLC. These results provide a comprehensive outline of frequently mutated genes and missense mutations that could be clinically actionable. These data also provide evidence of differing genomic landscapes between ever versus never smokers and primary NSCLC compared to the BM. This information could have important consequences for the selection and development of targeted drugs for these patients.

**Key Points**

- Reported genes and missense mutation in brain metastases (BMs) derived from non–small cell lung cancer could inform targeted treatment.
- Highlighting the discordance between BM and the primary tumor provides insight that treatment for the primary tumor may not be effective for the BM.

**Importance of the Study**

Brain metastases (BMs) derived from non–small cell lung cancer (NSCLC) represent a significant clinical problem. We provide a comprehensive systematic review of the genomic landscape of brain metastatic NSCLC to better inform novel precision medicine approaches. This review reports frequently mutated genes in BM derived from NSCLC and the most common missense mutations, with information on drug targets. Differing genomic profiles in NSCLC BM compared to the NSCLC primary and between smoking status are highlighted. Overall, this information could have important consequences for the selection and development of targeted drugs for patients.

Lung cancer causes more deaths worldwide (18.4%) than any other cancer type, leading to around 1.8 million deaths per year.^1^ Non–small cell lung cancer (NSCLC) represents around 80%–90% of lung cancers, with most patients presenting with advanced-stage unresectable disease,^2^ around 27% of patients will develop brain metastases (BMs).^3^ Major histological subtypes of NSCLC include adenocarcinoma (the most common subtype), squamous cell carcinoma (SCC), large cell carcinoma (LCC), adenosquamous carcinoma, and sarcomatoid carcinoma.^4^

The genetic landscape of the different subtypes of NSCLC is well established. *TP53* and *LRP1B* mutations are common to all NSCLC subtypes, but certain subtypes also have specific alterations. Lung adenocarcinoma has higher frequencies of *KRAS*, *EGFR*, *KEAP1*, *STK11*, *MET*, and *BRAF* somatic mutations. SCC shares many alterations with lung adenocarcinoma, but has specific somatic alterations including *TP53*, *LRP1B*, *CDKN2A*, *PTEN*, *PIK3CA*, *KEAP1*, *MLL2*, *HLA-A*, *NFE2L2*, *NOTCH1*, *RB1*, and *PDYN*.^4^ Some studies suggest the genomic landscape of NSCLC in ever versus never smokers differ independent of subtype. One study found *EGFR* mutations, *ROS1* and *ALK* fusions to be more prevalent in never smokers, whereas *KRAS, TP53, BRAF, JAK2, JAK3* and mismatch repair gene mutations were more commonly mutated in ever smokers.^5^

The profiles of BM derived from NSCLC are not as well evidenced. A recent large cohort study found *TP53*, *KRAS*, *CDKN2A*, *STK11*, *CDKN2B*, *EGFR*, *NKX2-1*, *RB1*, *MYC*, and *KEAP1* genes to be frequently mutated.^6^ This study also suggested different genomic profiles in the primary NSCLC compared to the BM.^6^

The recent emergence of targeted therapies to programmed cell death ligand 1 (PD-L1) has dramatically improved the survival of advanced NSCLC patients through targeting immune checkpoints to enhance tumor-directed immunity.^7^ Tumors with specific mutations may respond less well to immunotherapy drugs, and FDA-approved drugs that target specific mutations in *EGFR* and *ALK* may be more effective.^8^ These are now under investigation for patients with NSCLC BM but it is not clear whether selecting agents based on the mutation profile of the primary tumor is appropriate. New targeted therapies using agents with high CNS penetration that target appropriate mutations are also needed to improve the quality of life and survival for these patients.^9^ In this systematic review, we aim to collate genomic sequencing data of BM derived from NSCLC to identify commonly mutated genes and missense mutations, and assess their clinical actionability. We also aim to compare the genomic profile of ever versus never smokers, and primary NSCLC against the BM.

## Materials and Methods

### Protocol

We registered a protocol on the International Prospective Register of Systematic Reviews (PROSPERO: https://www.crd.york.ac.uk/prospero/display_record.php?ID=CRD42022321782) and followed the guidelines of Preferred Reporting Items for Systematic Reviews and Meta-Analyses (PRISMA).^10,11^ We did not require ethical approval for this study as all the data used in our analyses were from previously published articles.

### Search Strategy and Selection Criteria

We considered studies to be eligible if they (i) included samples/patients clinically diagnosed with a BM derived from NSCLC; (ii) had at least 2 mutations analyzed in the sequencing of BM; (iii) performed sequencing on BM tissue; and (iv) were cohort studies (including randomized trials and other controlled/uncontrolled clinical trials), case series, or case reports. There were no restrictions on language.

We identified records through a systematic literature search of Embase, MEDLINE, Web of Science, and BIOSIS from inception to 18/19 May (Supplementary Tables 1–4), we then uploaded the records to Endnote and de-duplicated.^12^ Next, we uploaded the remaining articles to Rayyan.^13^ Two independent reviewers screened records by title and abstract using Rayyan software and records which did not fit eligibility criteria were excluded. Two independent reviewers assessed the eligibility of the full texts for all remaining references. Any discrepancies during the screening process were referred to a third reviewer.

We carried out the data extraction into a Microsoft Excel document. We extracted data on the following: as publication details, patient characteristics, subtype of NSCLC, time to BM, overall survival, and genes mutated in BM. One reviewer extracted the data from each included record and a second reviewer checked this. We did not extract data looking at loss of heterozygosity. In addition to our prespecified data extraction, we extracted information on PD-L1 protein expression from the BM since this has emerged as an important biomarker for response to immune checkpoint inhibitors. Where the data were available, we also assessed if the primary NSCLC tumor had the same gene mutated as the BM, since this could provide important information regarding whether targeted treatment can be selected without access to BM tissue.

### Risk of Bias

One reviewer assessed risk of bias in the included studies using the Hoy et al. risk-of-bias tool.^14^ We considered studies to be at low risk of bias where all items received a yes response, moderate risk where 1 item received a no response, and high risk where 2 or more items received a no response.

### Statistical and Actionability Analysis

We synthesized the data from the included papers using Microsoft Excel, which we also used to create result tables and bar charts. We included a subgroup analysis looking at the genomic profile of BM in ever and never smokers, as defined in the individual publications. For all patients (including never- and ever-smoker subgroups), we also investigated distinct missense mutations present in frequently mutated genes. This analysis only included data that specified the exact type of missense mutation sequenced.

We used OncoKB to look at specific missense mutations found in the top 10 mutated genes in all patients to generate the level of evidence for each biomarker and considered if they could be actionable (https://www.oncokb.org).^15^ We also used the drug–gene interaction database (DGIdb) to assess the potential druggability of the selected genes (https://www.dgidb.org).^16^ We searched ClinicalTrials.gov to identify ongoing or completed clinical trials of drugs targeting mutant genes in NSCLC BM (https://clinicaltrials.gov). We refined our search by using the terms “brain metastasis,” “CNS,” “brain metastases,” “Non-small Cell Lung Cancer” and selecting for recruiting, active, not recruiting, completed studies, and only considering adults or older adults.

### Mutation Similarity Between Brain Metastasis and NSCLC Primary

We investigated the top 10 most commonly mutated genes in our gene list. We only included gene mutations that specified the distinct mutation in the BM and the primary. Copy number variant and other nonspecific mutations were not included. We identified the mutation in the NSCLC BM and then looked at the same gene in the primary tumor to see if there was the same/different/no mutation.

## Results

### Study Selection and Characteristics

**Figure 1.**
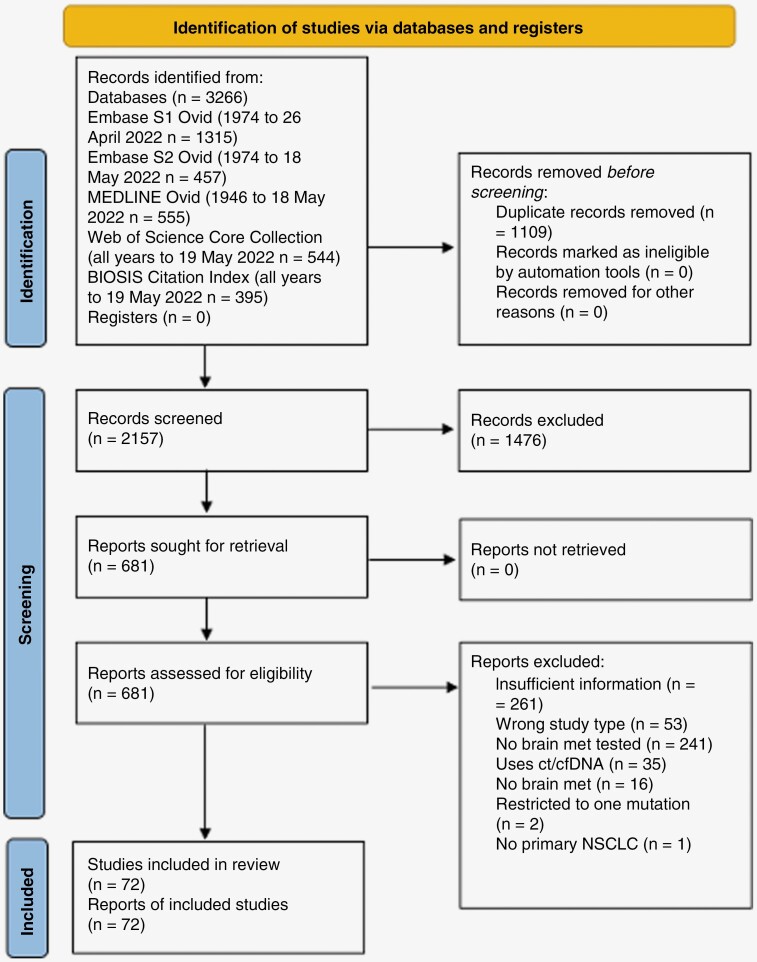
PRISMA diagram of included studies in genomic landscape of NSCLC-derived brain metastasis.

We carried out a systematic literature search on Embase, MEDLINE, Web of Science, and BIOSIS (number of papers, *n* = 3266) (Supplementary Tables 1–4). Of these papers, 1109 were duplicates, 1476 were excluded after title and abstract screen, and 609 were removed after full-text screen (Figure 1). A total of 72 distinct studies were included, with data on 2346 patients. Summary data were reported for 1798 of these patients and individual data for 567; some papers reported both (Table 1). We found 28 studies to be at low risk of bias, 31 at moderate risk, and 13 at high risk of bias (Supplementary Figures 1 and 2). We found 31 studies to have a high risk of bias due to not providing an acceptable case definition. For example, when a study stated the presence of a mutation, for example, mutated *EGFR*, but not the specific type of mutation, for example, L858R missense mutation in *EGFR* gene. So, we could not include these data in missense mutation analysis and the comparison between the genomic landscape of BM and primary NSCLC.

**Table 1.** Study characteristics of included studies.

Author	Country	Patients with non–small cell lung cancer brain metastasis		Female (%)	Age, years (range)	Primary NSCLC diagnosis	Median time between non–small cell lung cancer diagnosis and brain metastasis detection (months)	Overall survival (months)
		Total patients (*n* = 2346)	Matched pairs of primary cancer and brain metastasis (*n* = 588)					
Aljohani, H. M.^32^	USA	5	5	N/A	N/A	N/A	N/A	N/A
Balak, M. N.^33^	USA	1	1	100	72	Adenocarcinoma	N/A	N/A
Bekar, A.^34^	Turkey	26	0	3.8	Mean: 55.84 (21–78)	N/A	N/A	N/A
Brastianos, P. K.^35^	USA	33	33	66.7	N/A	Adenocarcinoma (88.2%), Squamous (11.8%)	Mean: 10.5	Mean: 25.2
Calles, A.^36^	USA	1	0	N/A	N/A	N/A	N/A	N/A
Chai, R. C.^37^	China	1	0	100	56	Adenocarcinoma	N/A	N/A
Cheok, S.^38^	USA	3	3	33.3	Mean: 67.33 (51–86)	N/A	N/A	N/A
Clay, T. D.^39^	N/A	2	0	50	Mean: 69 (55–83)	Adenocarcinoma (100%)	N/A	N/A
De Martino, L.^40^	Italy	1	0	100	10	Adenocarcinoma	0	23
Facchinetti, F.^41^	Italy	1	0	100	54	Adenocarcinoma	0	N/A
Ferguson, S. D.^42^	N/A	293	0	54.9	Median: 61	N/A	N/A	N/A
Fu, Z.^43^	China	1	1	100	38	Large cell neuroendocrine	0	10
Fukumura, K.^44^	USA	13	13	53.8	Mean: 61.6 (48–78)	Adenocarcinoma (69.2%), Squamous (30.8%)	Mean: 28.0	Mean: 15.4
Gautschi, O.^45^	N/A	1	1	100	56	N/A	N/A	N/A
Gow, C. H.^46^	Taiwan	12	12	40	Median: 61	Adenocarcinoma (83.3%), Squamous (16.7%)	N/A	N/A
Gow, C. H.^47^	Taiwan	1	1	100	48	Adenocarcinoma	23	9
Hadfield, M. J.^48^	USA	1	1	0	57	Adenocarcinoma	0	24
Harada, G.^49^	Brazil	1	1	0	53	Adenocarcinoma	5.1	6.5
Hermans, B. C. M.^50^	Netherlands	2	0	50	Mean: 48.5 (34–63)	Large cell neuroendocrine (100%)	N/A	Mean: 49.5
Huang, C. C.^51^	Taiwan	49	0	N/A	N/A	Adenocarcinoma (100%)	N/A	N/A
Illei, P. B.^52^	USA	1	0	N/A	38	Adenocarcinoma	N/A	N/A
Ito, N.^53^	Japan	1	0	100	71	Adenocarcinoma	N/A	5
Jafri, S. H. R.^54^	USA	1	0	100	52	Adenocarcinoma	0	7
Jiang, T.^55^	China	16	11	50	Mean: 50 (36–62)	Adenocarcinoma (100%)	N/A	N/A
Jiang, T.^56^	China	5	5	40	Mean: 52 (36–65)	Adenocarcinoma (100%)	N/A	N/A
Jing, W.^57^	China	1	1	100	52	Adenocarcinoma	6	22
Kamila, W. K.^58^	Poland	143	0	30.8	Median: 59	Adenocarcinoma (42.6%), squamous (16.1%), large cell (14.7%), non-other specified (NOS) (26.6%)	N/A	N/A
Kandioler, D.^59^	Austria	1	1	N/A	N/A	Adenocarcinoma	N/A	N/A
Kim, K. M.^60^	Korea	18	18	33.3	Mean: 60.1 (36–73)	N/A	N/A	N/A
Kudo, Y.^61^	USA	37	37	56.4	Median: 61 (40–84)	Adenocarcinoma (61.5%), squamous (20.5%), large cell (7.7%), and others (10.3%)	N/A	N/A
Leclair, N.^62^	USA	1	0	0	57	Adenocarcinoma	0	30
Lee, H. Y.^63^	Korea	3	0	N/A	N/A	N/A	N/A	N/A
Li, D.^17^	China	54	11	53.7	Mean: 55.6 (42–81)	Adenocarcinoma (100%)	N/A	N/A
Liao, L.^64^	China	6	6	16.7	Mean: 55.8 (33–67)	Adenocarcinoma (100%)	Mean: 9	N/A
Li, L.^65^	China	7	7	42.9	Mean: 50.1 (38–63)	Adenocarcinoma (100%)	Mean: 12.9	N/A
Liu, Z.^66^	China	12	12	50	N/A	Adenocarcinoma (66.7%), squamous (8.3%), large cell (8.3%), adenosquamous (8.3%), clear cell, and tubular adenocarcinoma (8.3%)	N/A	Mean: 37.4
Luo, D.^67^	China	136	14	39	Median: 55 (26–79)	Adenocarcinoma (82.4%), squamous (3.7%), adenosquamous carcinoma (5.9%), large cell carcinoma (8.1%)	N/A	N/A
Ma, C.^68^	China	5	0	0	N/A	N/A	N/A	N/A
Ma, Y.^69^	China	15	0	53.3	Median: 55 (Range: 35–65)	Adenocarcinoma (100%)	N/A	N/A
Martinez-Marti, A.^70^	Spain	2	2	50	Mean: 59 (44–74)	Adenocarcinoma (100%)	N/A	N/A
Martinez-Marti, A.^71^	Spain	1	1	N/A	N/A	Adenocarcinoma	0	36
Nayyar, N.^72^	USA	73	0	67.1	N/A	Adenocarcinoma (100%)	N/A	N/A
Nicos, M.^73^	Poland	150	0	32	59.8 (38–81)	Adenocarcinoma (44%), squamous (16%), giant cell (15%), not otherwise specified (25%)	N/A	N/A
Nicos, M.^74^	Poland	145	0	31	Median: 60	Adenocarcinoma (55.2%), squamous (20%), large cell (15.1%), not otherwise specified (9.7%)	N/A	Median: 13.5
Ogata, M.^75^	Japan	1	1	100	64	Adenocarcinoma	24	N/A
Patil, T.^76^	USA	1	1	0	40	Squamous	0	74
Powrozek, T.^77^	Poland	143	0	30.8	Mean: 59.8 (38–81)	Adenocarcinoma (42.7%), squamous (16.1%), large cell (14.7%), not otherwise specified (26.6%)	N/A	Median: 9.2
Preusser, M.^78^	Austria	76	0	32.9	Mean: 57.3 (38–78)	Adenocarcinoma (100%)	Median: 0	Median: 13.5
Rau, K. M.^79^	Taiwan	49	49	44.9	Mean: 64 (46–86)	Adenocarcinoma (100%)	N/A	N/A
Sakakibara-Konishi, J.^80^	Japan	1	0	100	40	Adenocarcinoma	N/A	N/A
Saunus, J. M.^81^	Australia	18	0	27.8	N/A	Adenocarcinoma (66.7%), Adenosquamous (11.1%), squamous (11.1%), large cell (11.1%)	Mean: 19.8	Mean: 32.8
Schaettler, M. O.^82^	USA	5	0	60	Mean: 64.8 (54–81)	N/A	N/A	N/A
Schlegel, U.^83^	USA	5	5	16.7	N/A (47–72)	Adenocarcinoma (66.7%) and large cell (33.3%)	N/A	N/A
Shan, C. G.^84^	China	1	0	0	44	Adenocarcinoma	2	14
Song, Z.^18^	China	27	27	33.3	N/A	Adenocarcinoma (77.8%), squamous (22.2%)	N/A	N/A
Stein, M. K.^85^	USA	143	0	57	Median: 64 (31–84)	Adenocarcinoma (100%)	N/A	N/A
Stella, G.^86^	Italy	68	68	N/A	N/A	Adenocarcinoma (54.4%), Squamous (20.5%), Neuroendocrine (10.2%) and undifferentiated (14.9%)	N/A	N/A
Sun, M.^87^	USA	55	55	35	N/A	Adenocarcinoma (73%), squamous (23%), large cell (2%), adenosquamous (2%)	Median: 14.8	N/A
Tafe, L. J.^88^	USA	31	0	58	Median: 70 (51–89)	Adenocarcinoma (77.4%), squamous (22.6%)	N/A	N/A
Talreja, V.^89^	India	1	0	0	47	Squamous	N/A	N/A
Tseng, L. H.^90^	USA	12	9	N/A	N/A	N/A	N/A	N/A
Vassella, E.^91^	Switzerland	56	56	N/A	N/A	N/A	N/A	N/A
Villaruz, L. C.^92^	USA	200	38	N/A	N/A	N/A	N/A	N/A
Wang, H.^93^	China	61	61	29.5	Mean: 55.5 (29–74)	Adenocarcinoma (82.0%), mixed (13.1%), squamous (4.9%)	Mean: 17.0	N/A
Wang, W.^94^	China	1	0	0	48	Adenocarcinoma	0	N/A
Wu, H.^19^	China	1	0	100	50	N/A	0	N/A
Wu, P. F.^95^	Taiwan	86	0	48	Median: 59 (29–82)	Adenocarcinoma (100%)	Mean: 6.3	N/A
Xu, Y.^96^	China	18	18	N/A	N/A	N/A	N/A	N/A
Yan, J.^97^	China	1	0	100	27	Adenocarcinoma	2	90
Yang, J.^98^	China	1	1	0	62	Adenocarcinoma	0	23
Zhou, Y.^99^	USA	1	0	N/A	N/A	Adenocarcinoma	N/A	N/A
Zhu, W.^100^	USA	1	1	100	51	Adenocarcinoma	N/A	N/A

The majority of patients with individual-level data included in this analysis were histologically diagnosed with adenocarcinoma (*n* = 387, 67.2%), SCC (*n* = 35, 6.1%), adenosquamous carcinoma (*n* = 15, 2.6%), and LCC (*n* = 11, 1.9%). The rest were unknown, or the data were unavailable. This is similar to the NSCLC population demographic; however, there is a slight overrepresentation of the adenocarcinoma subtype. Overall, from the limited demographic data we have, we expect these to follow the typical NSCLC population demographic (Table 1).

The most prevalent sequencing techniques in our cohort were the following; next-generation sequencing (NGS; *n* = 690, 29.41%), multiple techniques (*n* = 500, 21.31%), Sanger sequencing (*n* = 193, 8.23%), EGFR mutation kit (*n* = 136, 5.80%), and whole exome sequencing (*n* = 112, 4.77%). There were 4r studies which did not report specific methods (*n* = 221, 9.42%).

We observed 4 patients who had >600 mutations reported, so we initially did not extract the data. Once we discovered the gene list of >25 mutations in NSCLC BM, we checked to see if these 4 patients had the same mutated gene, and if so, this was added to the analysis. We also identified 3 patients with >5 mutations in a single gene; this was reported as only 5 mutations to avoid outlier bias.

### Frequently Mutated Genes

**Figure 2.**
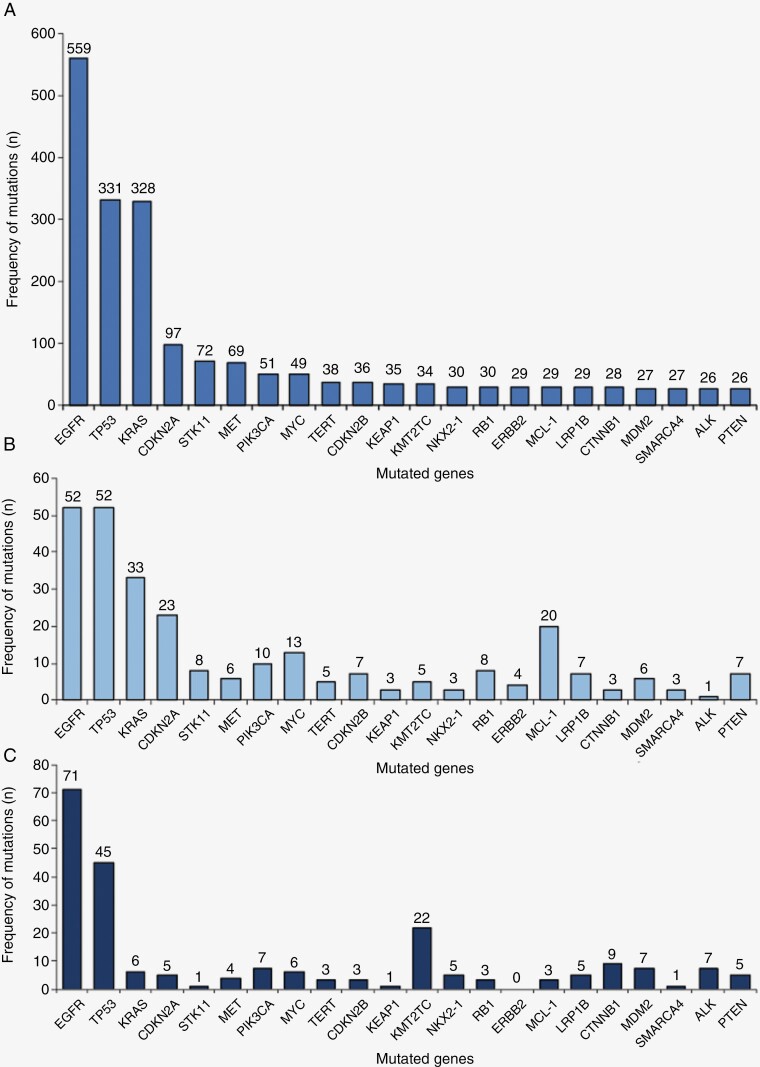
Common mutated genes within the NSCLC BM cohort in decreasing order. (A) All patients, (B) ever smokers, (C) never smokers.

We found over 350 genes to be mutated at least twice in NSCLC BM. A total of 22 genes had >25 mutations across the included studies: *EGFR* (number of mutations, *n* = 559), *TP53* (*n* = 331), *KRAS* (*n* = 328), *CDKN2A* (*n* = 97), *STK11* (*n* = 72), *MET* (*n* = 69), *PIK3CA* (*n* = 51), *MYC* (*n* = 49), *TERT* (*n* = 38), *CDKN2B* (*n* = 36), *KEAP1* (*n* = 35), *KMT2C* (*n* = 34), *NKX2-1* (*n* = 30), *RB1* (*n* = 30), *ERBB2* (*n* = 29), *MCL-1* (*n* = 29), *LRP1B* (*n* = 29), *CTNNB1* (*n* = 28), *MDM2* (*n* = 27), *SMARCA4* (*n* = 27), *ALK* (*n* = 26) and *PTEN* (*n* = 26) (Figure 2A).

We further subgrouped our NSCLC BM cohort to never and ever smokers. Most papers did not report individual smoking status, so this analysis only included a total of 115 ever smokers and 114 never smokers (Figure 2B and 2C). The top 5 mutated genes in ever smokers were *TP53* (*n* = 52), *EGFR* (*n* = 52), *KRAS* (*n* = 33), *CDKN2A* (*n* = 23), *MCL-1* (*n* = 20), *MYC* (*n* = 13) and *PIK3CA* (*n* = 10) (Supplementary Figure 3A). For the never smokers, *EGFR* (*n* = 71), *TP53* (*n* = 45), *KMT2C* (*n* = 22), *NOTCH2* (*n* = 12), and *CTNNB1* (*n* = 9) were most frequently mutated (Supplementary Figure 3B).

### Distinct Missense Mutations

For the top 10 mutated genes (*TP53*, *EGFR*, *KRAS*, *CDKN2A*, *STK11*, *MET*, *PIK3CA*, *MYC*, *TERT*, and *CDKN2B*), we further investigated each distinct missense mutation reported. *TP53* had a wide range of distinct missense mutations with a total of 74. Only 11.6% (*n* = 10) of studies reported more than one of the same mutations, with the most common mutations R248L and V157F mutated 3 times (3.5%) (Supplementary Figure 4). For *EGFR*, there were 25 distinct missense mutations, most of these mutations were L858R 67.8% (*n* = 80), T790M 6.8% (*n* = 8), and G719S 4.2% (*n* = 5) (Supplementary Figure 5). *KRAS* was found to have 12 distinct missense mutations, these included G12C 30.4% (*n* = 17), G12V 16.1% (*n* = 9), and G13C 14.3% (*n* = 8) (Supplementary Figure 6). *CDKN2A* had a total of 5 distinct missense mutations, with the most common being V115L 33.3% (*n* = 2) (Supplementary Figure 7). *STK11* had 7 distinct missense mutations, each mutated once (Supplementary Figure 8). *MET* only had a single specific missense mutation reported which was G1146A. *PIK3CA* was found to have 7 distinct missense mutations and the most common being E545K 38.5% (*n* = 5) (Supplementary Figure 9). *MYC* had 4 specific missense mutations, with each mutated once (Supplementary Figure 10). *TERT* had 2 distinct missense mutations (P259L and R622H) both only mutated once. *CDKN2B* had no specified missense mutations with most of the mutations relating to copy number variation (CNV).

We further looked at the distinct missense mutations of *TP53* and *EGFR* in BM of ever and never smokers. *TP53* had no concordant missense mutations between ever versus never smokers (Supplementary Figure 11). For *EGFR*, ever and never smokers had 7 and 8 L858R mutations, respectively. Both groups were found to have 2 T790M mutations, but no other concordant mutations were found (Supplementary Figure 12).

### Clinically Actionable Mutations and Drugs

For our commonly mutated gene list in all patients, DGIdb found 22 clinically actionable genes, 15 genes related to drug resistance, and 13 that have a potentially druggable genome (Supplementary Table 5). Of 91 studies identified in the clinical trial search, 38 were of drugs to target mutated genes (Supplementary Table 6).

### Biomarker Evidence and FDA-Approved Drugs

L858R, T790M, G719, and L861Q *EGFR* missense mutations and G12C *KRAS* missense mutation are FDA-recognized biomarkers predictive of response to an FDA-approved drug (level 1) reported in NSCLC (Table 2). For *EGFR,* afatinib targets L858R, G719, and L861Q, osimertinib targets L858R and T790M, dacomitinib, erlotinib, erlotinib + ramucirumab combination, and gefitinib target L858R. For *KRAS*, adagrasib and sotorasib target G12C. Osimertinib has been FDA approved for targeting G719 and L861Q, and these are currently standard-of-care biomarkers (level 2). Other drugs have been considered for missense mutations in *TP53*, *EGFR*, *KRAS*, *CDKN2A*, *STK11*, and *PIK3CA* but these are not FDA approved (Table 2). For EGFR, T790M is a standard-of-care biomarker predictive of resistance to erlotinib, gefitinib, and afatinib in NSCLC, D761Y is also considered a biomarker of resistance to gefitinib but this is less well evidenced (Table 2 and Supplementary Figure 12). It is important to note these levels of biomarker evidence have been accepted for systemic therapies (solid tumors and NSCLC), but this is not evidenced in BM (Table 2).

**Table 2.** Level of evidence for drugs targeting missense mutations in NSCLC and all solid tumors for the missense mutations NSCLC BM cohort found on OncoKB

Gene	Missense mutation	Level of evidence	Drugs	Level-associated cancer types
*TP53*	Y220C	3A	PC14586	All solid tumors
*EGFR*	L858R, exon-19 in-frame deletions	1	Afatinib Dacomitinib Erlotinib Erlotinib + Ramucirumab Gefitinib Osimertinib	NSCLC
	L858R, S768I, G719, L861Q, exon-19 in-frame deletions, exon-19 in-frame insertions	3A	Patritumab Deruxtecan	NSCLC
	T790M	1	Osimertinib	NSCLC
		R1	Erlotinib Gefitinib Afatinib	
	G719	1	Afatinib	NSCLC
		2	Osimertinib	
	L861Q	1	Afatinib	NSCLC
		2	Osimertinib	
	D761Y	4	Osimertinib	NSCLC
		R2	Gefitinib	
	L747P	4	Afatinib	NSCLC
*KRAS*	G12C	1	Adagrasib	NSCLC
		1	Sotorasib	
		4	Trametinib Cobimetinib Binimetinib	All solid tumors
	G12V			
	G13C			
	Q61H			
	G13D			
	G12A			
	G12F			
	G12S			
	P34L			
	Q61L			
	G12D			
	G12D	4	RMC-6236	All solid tumors
*CDKN2A*	Oncogenic mutations	4	Abemaciclib Palbociclib Ribociclib	All solid tumors
*STK11*	H174R	4	Bemcentinib + Pembrolizumab	NSCLC
	E223V			
*PIK3CA*	E545K	4	RLY-2608	All solid tumors
	E542K			
	G118D			
	Q546K			
	H1047R			
		4	LOXO-783	All solid tumors

Level 1 = FDA-recognized biomarker predictive of response to an FDA-approved drug in this indication, level 2 = standard care biomarker to an FDA-approved drug in this indication, level 3A = compelling clinical evidence biomarker is predictive of response to drug in this indication, level 3B = standard care or investigational biomarker predictive of response to FDA-approved or investigational drug in another indication, level 4 = compelling biological evidence biomarker is predictive of response to a drug. Level R1 = standard care biomarker predictive of resistance to an FDA-approved drug in this indication, level R2 = compelling clinical evidence biomarker is predictive of resistance to a drug.

### Mutation Similarity Between BM and NSCLC

There were 647 mutations among the top 10 overall mutated genes incorporated in this analysis. We identified 408 mutations (63%) which were the same in both the BM and the primary NSCLC, and 239 mutations (37%) that were discordant. Of this subgroup, *TP53* (*n* = 121), *EGFR* (*n* = 94), and *KRAS* (*n* = 65) have the most data. We found the mutations that were most often similar between BM and NSCLC were in *TP53* (67%), *KRAS* (66%), and *EGFR* (58%).

### PD-L1 Expression

We identified the percentage of PD-L1 expression in the BM, although these were only reported in three of the 72 included studies.^17–19^ We found a total of 28 patients, consisting of 21 lung adenocarcinoma (75%), 6 SCC (21.4%), and 1 with subtype data unavailable (3.6%). Of this subgroup, 25 patients (89.3%) were found to have 0%–49% of PD-L1 expression. Only 3 patients (10.7%) had PD-L1 expression which was >50% and these patients were all diagnosed with lung adenocarcinoma (Table 3). Patients are classified as having a high PD-L1 expression if a tumor proportion score ≥50%, as this is the FDA-approved level for first-line treatment of primary NSCLC.^20^

**Table 3.** PD-L1 expression in patients included in the NSCLC brain metastasis cohort

NSCLC subtype	PD-L1 expression in brain metastasis (%)
Lung adenocarcinoma	50–100
Lung adenocarcinoma	50–100
Lung adenocarcinoma	65
Lung adenocarcinoma	1–49
Lung adenocarcinoma	1–49
Lung adenocarcinoma	1–49
Lung adenocarcinoma	1–49
Lung adenocarcinoma	1–49
Lung adenocarcinoma	40
Lung adenocarcinoma	<1
Lung adenocarcinoma	0
Lung adenocarcinoma	0
Lung adenocarcinoma	0
Lung adenocarcinoma	0
Lung adenocarcinoma	0
Lung adenocarcinoma	0
Lung adenocarcinoma	0
Lung adenocarcinoma	0
Lung adenocarcinoma	0
Lung adenocarcinoma	0
Lung adenocarcinoma	0
Squamous cell carcinoma	1–49
Squamous cell carcinoma	1–49
Squamous cell carcinoma	1–49
Squamous cell carcinoma	0
Squamous cell carcinoma	0
Squamous cell carcinoma	0
N/A	<1

## Discussion

This review included 72 studies with data from 2346 patients with BM derived from NSCLC, of which 567 had individual-level data. These studies provided information on the commonly mutated genes and missense mutations in BM derived from NSCLC, comparison of the genomic landscape between ever versus never smokers and primary NSCLC versus BM, and PD-L1 expression in BM.

In our cohort, over 350 genes were reported to be mutated at least twice, with 22 genes found to have >25 mutations. Twelve of these mutated genes were found to be concordant with a large cohort study of BM from NSCLC: *EGFR*, *TP53*, *KRAS*, *CDKN2A*, *STK11*, *PIK3CA*, *MYC*, *CDKN2B*, *KEAP1*, *NKX2-1*, *SMARCA4*, and *RB1* (Figure 2A).^6^ The same study also found *NFKBIA*, *RICTOR*, and *NF1* to be frequently mutated; these genes were also identified in our cohort but were not in our top mutated genes.^6^ A meta-analysis found *TP53*, *EGFR*, *KRAS*, *STK11*, and *EML4-ALK* to be frequently mutated in NSCLC.^21^ We identified a similar pattern in our BM derived from NSCLC. However, our study discovered some differences between the mutations present in the primary NSCLC and the BM. Primary NSCLC and BM were found to harbor different mutations in 37% of cases, this evidence is in keeping with previous studies suggesting the NSCLC primary and derived BM suggest genetic differences, thus highlighting the importance of sequencing BM derived from NSCLC due to differing genomic landscapes.^6,22^

The frequently mutated genes in BM derived from NSCLC included *TP53*, *EGFR*, *KRAS*, *CDKN2A*, *STK11*, *MET*, *PIK3CA*, *MYC*, *TERT*, and *CDKN2B*, which we considered to be of most interest to target for intervention. Currently, *EGFR* and *ALK* have the most well-established actionable genetic alterations for metastases derived from NSCLC. EGFR has 3 generations of treatment including gefitinib and erlotinib (first generation), afatinib and dacomitinib (second generation), and osimertinib (third generation).^8,23–27^ These drugs were also identified in our OncoKB database search with varying levels of biomarker evidence depending on the mutation type. *ALK* also presented many treatment options such as alectinib, although this was less frequently mutated in our gene list.^8,28^ More recently, drugs have been discovered that target genes that were previously difficult, such as *KRAS*. Two G12C inhibitors have been approved (sotorasib and adagrasib), with other clinical trials ongoing.^8^ OncoKB identified a number of drugs that are currently being tested in our frequently mutated gene list, but these are not FDA approved. These drugs included *TP53* with PC14586 in all solid tumors, *EGFR* with patritumab deruxtecan in NSCLC, *KRAS* with trametinib, cobimetinib, and binimetinib in all solid tumors, *CDKN2A* with abemaciclib, palbociclib, and ribociclib, *STK11* with bemcentinib + pembrolizumab, and *PIK3CA* with RLY-2608 and LOXO-783 in all solid tumors (Table 2).^15^

In our smoking subgroup analysis, the genomic profile of BM in never smokers identified more *EGFR* mutations compared to ever smokers. Likewise, ever smokers had more *TP53* mutations. The genomic landscape comparing smoking status in BM seemed to differ, with alternative genes found to be frequently mutated, excluding *TP53* and *EGFR* (Figure 2B and 2C). Distinct missense mutations in *TP53* and *EGFR* between ever smokers were compared with never smokers and were found to differ, with the exception of L858R and T790M which were identified at similar frequencies. Interestingly, only ever smokers were found to have the missense mutations L861Q and G179S in *EGFR* which are clinically actionable (Table 2 and Supplementary Figure 12). Previous studies investigating the genomic landscape of NSCLC in ever versus never smokers found a similar pattern to our data, with *EGFR* mutations more frequent in never smokers, and *TP53* and *KRAS* more commonly mutated in ever smokers.^5^

Our data found high PD-L1 expression (>50%) to be uncommon in our cohort, with 25 patients (89.3%) with 0%–49% of PD-L1 expression and only 3 patients (10.7%) had >50% PD-L1 expression, suggesting that immune checkpoint inhibition may be effective in only a small proportion of these patients. PD-L1 was also found to be infrequently expressed in the BM in a previous study with found seven (21.9%) of patients with PD-L1 ≥5% and 25 (78.1%) of patients with PD-L1 <5%.^29^

There were some limitations to this review. We only included studies of patients/samples with sequenced tumor tissue rather than circulating tumor DNA as tissue sequencing is still the gold-standard technique for molecular tests.^30^ However, the consequence of this is that the many studies that sequence circulating tumor DNA were not included in our review. The data are also biased to BM where the brain tumor was resected, making tumor tissue available to sequence, which likely depends on both BM size and location.^31^ One limitation of the published literature is the lack of granularity on the lineage of the metastatic NSCLC, that is, adenocarcinoma versus SCC, and we would recommend that all subsequent genomic studies include precise diagnosis by lung pathologists, where possible.

Some studies we reviewed reported the presence of a mutation in a gene but did not clarify the specific type of mutation, so we could not include these data in our analysis of distinct missense mutations in top mutated genes in BM from NSCLC. In addition, for many of the studies using NGS and other sequencing platforms, we have no knowledge of genes that were not mutated as we did not have access to the full list of genes that were tested and/or which of those tests had failed. There also could be publication and reporting bias as candidate genes that are already known to be mutated in the NSCLC primary tumor are more likely to be sequenced, so their mutation status is more likely to be reported compared to lesser-known genes. Considering these limitations, we were not able to generate a prevalence estimate for each gene in the BM derived from NSCLC. The studies included in our review used a wide range of sequencing panels which may lead to some mutations being more represented or identified compared to others, which could have led to bias in our results. There is also a slight overrepresentation of adenocarcinoma in the NSCLC population in our cohort, which may lead to bias with mutations commonly seen in this subtype to be identified more frequently.

The genomic landscape of BM compared to the NSCLC primary should be interpreted with caution as our search criteria identified BM which had a mutation and we then looked to see if the same gene was mutated in the primary NSCLC. Therefore, the data is biased toward BM gene mutations, as we are missing the data where the primary NSCLC has a mutated gene that is not identified in the BM. In this analysis, we were also unable to include mutations that were identified in either primary or BM but which lacked an exact description to define if they matched, that is, when the gene has a missense mutation versus L858R missense mutation, the first option was insufficient. Similarly, we were not able to include CNV variation in this analysis as we were unable to identify the number of copies of each gene that were present.

## Conclusions

To our knowledge, this is the first systematic review that assessed the genomic landscape of BM derived from NSCLC. We highlight the most frequently mutated genes (*TP53*, *EGFR*, *KRAS*, *CDKN2A*, and *STK11*) and most frequently reported missense mutations (L858R in *EGFR* and G12C in *KRAS*) in BM derived from NSCLC, and assessed their potential clinical actionability. Moreover, we found gene mutations in NSCLC BM to differ compared to the NSCLC primary. We also identified different genomic profiles in BM of ever versus never smokers. These differences could have important implications for the selection and development of targeted agents for these patients.

## Supplementary Material

vdad145_suppl_Supplementary_Material

vdad145_suppl_Supplementary_Data
