# Supplementary material for: Genomic landscape and actionable mutations of brain metastases derived from non–small cell lung cancer: A systematic review
Source: Neurooncol Adv. 2023 Nov 24;5(1):vdad145. doi: 10.1093/noajnl/vdad145 (PMC10734675; doi:10.1093/noajnl/vdad145)
Supplement: vdad145_suppl_Supplementary_Material [file vdad145_suppl_supplementary_material.docx]

| **Ovid Embase <1974 to 2022 May 18>** | | |
| --- | --- | --- |
| 1 | exp LUNG TUMOUR/ | n=430492 |
| 2 | ((lung or pulmonary or bronch*) and (cancer* or carcinoid* or tumour* or tumor* or carcinoma* or adenocarcinoma* or adenoma* or granuloma* or sarcoma* or neoplas*)).mp. | n=788296 |
| 3 | NSCLC.mp. | n=99065 |
| 4 | lung metastasis/ | n=58632 |
| 5 | ((lung or pulmonary or bronch*) adj3 metasta*).mp. | n=88818 |
| 6 | or/1-5 | n=793437 |
| 7 | exp BRAIN TUMOR/ | n=190814 |
| 8 | ((brain or cerebr* or intracebr* or crani* or intracrani* or midline or mening* or leptomening* or subtentorial or sub-tentorial or supratentorial or supra-tentorial) and (cancer* or carcinoid* or tumour* or tumor* or carcinoma* or adenocarcinoma* or adenoma* or granuloma* or sarcoma* or neoplas*)).mp. | n=468935 |
| 9 | (cerebroma* or encephalophyma*).mp. | n=49 |
| 10 | BRAIN METASTASIS/ or meningeal metastasis/ | n=40074 |
| 11 | central nervous system metastasis/ and exp brain/ | n=399 |
| 12 | ((brain or cerebr* or intracebr* or crani* or intracrani* or midline or mening* or leptomening* or subtentorial or sub-tentorial or supratentorial or supra-tentorial) adj3 metasta*).mp. | n=51585 |
| 13 | or/7-12 | n=505692 |
| 14 | METASTASIS/ or distant metastasis/ | n=348820 |
| 15 | metasta*.mp. | n=1005218 |
| 16 | (secondary adj (cancer* or carcinoid* or tumour* or tumor* or carcinoma* or adenocarcinoma* or adenoma* or granuloma* or sarcoma* or neoplas*)).mp. | n=8315 |
| 17 | (disseminated adj (cancer* or carcinoid* or tumour* or tumor* or carcinoma* or adenocarcinoma* or adenoma* or granuloma* or sarcoma* or neoplas*)).mp. | n=6112 |
| 18 | ((cancer* or carcinoid* or tumour* or tumor* or carcinoma* or adenocarcinoma* or adenoma* or granuloma* or sarcoma* or neoplas*) adj (migration or spread*)).mp. | n=8065 |
| 19 | or/14-18 | n=1013891 |
| 20 | 6 and 13 and 19 | n=37063 |
| 21 | exp sequence analysis/ | n=378980 |
| 22 | sequencing.af. | n=599192 |
| 23 | sequences.ti. | n=44472 |
| 24 | sequences.ab. /freq=2 | n=145865 |
| 25 | (sequenc* and (FISH or (fluorescence adj3 hybridization) or immunohistochem* or immuno-histochem* or PCR or polymerase chain reaction*)).tw,kf. | n=280788 |
| 26 | (sequence* adj7 (amplification* or analys* or annotat* or array* or microarray* or assay* or data* or deletion* or determin* or genom* or genetic* or gene or genes or molecular or oncogen* or DNA or RNA or mRNA or miRNA or mutat* or mutant* or protein* or proteomic* or transcriptomic*)).tw,kf. | n=652188 |
| 27 | or/21-26 | n=1292002 |
| 28 | 20 and 27 | n=1752 |

Supplementary Table 1: Embase search terms and strategy.

| **Ovid MEDLINE(R) ALL <1946 to 2022 May 18>** | | |
| --- | --- | --- |
| 1 | exp Lung Neoplasms/ | n=260664 |
| 2 | ((lung or pulmonary or bronch*) and (cancer* or carcinoid* or tumour* or tumor* or carcinoma* or adenocarcinoma* or adenoma* or granuloma* or sarcoma* or neoplas*)).mp. | n=473848 |
| 3 | NSCLC.mp. | n=53922 |
| 4 | ((lung or pulmonary or bronch*) adj3 metasta*).mp. | n=43704 |
| 5 | or/1-4 | n=476311 |
| 6 | exp Brain Neoplasms/ | n=165175 |
| 7 | ((brain or cerebr* or intracebr* or crani* or intracrani* or midline or mening* or leptomening* or subtentorial or sub-tentorial or supratentorial or supra-tentorial) and (cancer* or carcinoid* or tumour* or tumor* or carcinoma* or adenocarcinoma* or adenoma* or granuloma* or sarcoma* or neoplas*)).mp. | n=311414 |
| 8 | (cerebroma* or encephalophyma*).mp. | n=56 |
| 9 | ((brain or cerebr* or intracebr* or crani* or intracrani* or midline or mening* or leptomening* or subtentorial or sub-tentorial or supratentorial or supra-tentorial) adj3 metasta*).mp. | n=23800 |
| 10 | or/6-9 | n=336339 |
| 11 | exp Neoplasm Metastasis/ | n=218256 |
| 12 | metasta*.mp. | n=649449 |
| 13 | (secondary adj (cancer* or carcinoid* or tumour* or tumor* or carcinoma* or adenocarcinoma* or adenoma* or granuloma* or sarcoma* or neoplas*)).mp. | n=5519 |
| 14 | (disseminated adj (cancer* or carcinoid* or tumour* or tumor* or carcinoma* or adenocarcinoma* or adenoma* or granuloma* or sarcoma* or neoplas*)).mp. | n=3397 |
| 15 | ((cancer* or carcinoid* or tumour* or tumor* or carcinoma* or adenocarcinoma* or adenoma* or granuloma* or sarcoma* or neoplas*) adj (migration or spread*)).mp. | n=5852 |
| 16 | or/11-15 | n=666031 |
| 17 | 5 and 10 and 16 | n=14816 |
| 18 | exp *genetic techniques/ | n=428923 |
| 19 | exp Sequence Analysis/ | n=440254 |
| 20 | sequencing.af. | n=395554 |
| 21 | sequences.tw,kf. | n=444693 |
| 22 | (sequenc* and (FISH or (fluorescence adj3 hybridization) or immunohistochem* or immuno-histochem* or PCR or polymerase chain reaction*)).tw,kf. | n=220330 |
| 23 | (sequence* adj7 (amplification* or analys* or annotat* or array* or microarray* or assay* or data* or deletion* or determin* or genom* or genetic* or gene or genes or molecular or oncogen* or DNA or RNA or mRNA or miRNA or mutat* or mutant* or protein* or proteomic* or transcriptomic*)).tw,kf. | n=601383 |
| 24 | 18 or 19 or 20 or 21 or 22 or 23 | n=1545758 |
| 25 | 17 and 24 | n=555 |

Supplementary Table 2: MEDLINE search terms and strategy.

| **Web of Science – Core collection <all years to 2022 May 19>** | | |
| --- | --- | --- |
| #1 | TS=(((lung or pulmonary or bronch*) NEAR (cancer* or carcinoid* or tumour* or tumor* or carcinoma* or adenocarcinoma* or adenoma* or granuloma* or sarcoma* or neoplas*)) or NSCLC or ((lung or pulmonary or bronch*) NEAR metasta*)) | n=455259 |
| #2 | TS=(((brain or cerebr* or intracebr* or crani* or intracrani* or midline or mening* or leptomening* or subtentorial or sub-tentorial or supratentorial or supra-tentorial) NEAR (cancer* or carcinoid* or tumour* or tumor* or carcinoma* or adenocarcinoma* or adenoma* or granuloma* or sarcoma* or neoplas*)) OR (cerebroma* or encephalophyma*) or ((brain or cerebr* or intracebr* or crani* or intracrani* or midline or mening* or leptomening* or subtentorial or sub-tentorial or supratentorial or supra-tentorial) NEAR metasta*)) | n=159553 |
| #3 | TS=(metasta* or “secondary cancer*” or “secondary carcinoid*” or “secondary tumour*” or “secondary tumor*” or “secondary carcinoma*” or “secondary adenocarcinoma*” or “secondary adenoma*” or “secondary granuloma*” or “secondary sarcoma*” or “secondary neoplas*” or “disseminated cancer*” or “disseminated carcinoid*” or “disseminated tumour*” or “disseminated tumor*” or “disseminated carcinoma*” or “disseminated adenocarcinoma*” or “disseminated adenoma*” or “disseminated granuloma*” or “disseminated sarcoma*” or “disseminated neoplas*” or ((cancer* or carcinoid* or tumour* or tumor* or carcinoma* or adenocarcinoma* or adenoma* or granuloma* or sarcoma* or neoplas*) NEAR/2 (migration or spread*))) | n=732708 |
| #4 | TS=(sequencing or sequences or ((sequence* AND (amplification* or analys* or annotat* or array* or microarray* or assay* or data* or deletion* or determin* or genom* or genetic* or gene or genes or molecular or oncogen* or DNA or RNA or mRNA or miRNA or mutat* or mutant* or protein* or proteomic* or transcriptomic*)) or (sequenc* AND (FISH or “fluorescence in situ hybridization” or immunohistochem* or immuno-histochem* or PCR or “polymerase chain reaction*”)))) | n=2100678 |
| #5 | (#1 and #2 and #3 and #4) | n=544 |

Supplementary Table 3: Web of Science search terms and strategy.

| **BIOSIS Citation Index <all years to 2022 May 19>** | | |
| --- | --- | --- |
| #1 | TS=(((lung or pulmonary or bronch*) AND (cancer* or carcinoid* or tumour* or tumor* or carcinoma* or adenocarcinoma* or adenoma* or granuloma* or sarcoma* or neoplas*)) or NSCLC or ((lung or pulmonary or bronch*) AND metasta*)) | n=475646 |
| #2 | TS=(((brain or cerebr* or intracebr* or crani* or intracrani* or midline or mening* or leptomening* or subtentorial or sub-tentorial or supratentorial or supra-tentorial) AND (cancer* or carcinoid* or tumour* or tumor* or carcinoma* or adenocarcinoma* or adenoma* or granuloma* or sarcoma* or neoplas*)) OR (cerebroma* or encephalophyma*) or ((brain or cerebr* or intracebr* or crani* or intracrani* or midline or mening* or leptomening* or subtentorial or sub-tentorial or supratentorial or supra-tentorial) AND metasta*)) | n=231976 |
| #3 | TS=(metasta* or “secondary cancer*” or “secondary carcinoid*” or “secondary tumour*” or “secondary tumor*” or “secondary carcinoma*” or “secondary adenocarcinoma*” or “secondary adenoma*” or “secondary granuloma*” or “secondary sarcoma*” or “secondary neoplas*” or “disseminated cancer*” or “disseminated carcinoid*” or “disseminated tumour*” or “disseminated tumor*” or “disseminated carcinoma*” or “disseminated adenocarcinoma*” or “disseminated adenoma*” or “disseminated granuloma*” or “disseminated sarcoma*” or “disseminated neoplas*” or ((cancer* or carcinoid* or tumour* or tumor* or carcinoma* or adenocarcinoma* or adenoma* or granuloma* or sarcoma* or neoplas*) NEAR/2 (migration or spread*))) | n=420573 |
| #4 | TS=(sequencing or sequences or ((sequence* AND (amplification* or analys* or annotat* or array* or microarray* or assay* or data* or deletion* or determin* or genom* or genetic* or gene or genes or molecular or oncogen* or DNA or RNA or mRNA or miRNA or mutat* or mutant* or protein* or proteomic* or transcriptomic*)) or (sequenc* AND (FISH or “fluorescence in situ hybridization” or immunohistochem* or immuno-histochem* or PCR or “polymerase chain reaction*”)))) | n=1702139 |
| #5 | (#1 and #2 and #3 and #4) | n=395 |

Supplementary Table 4: BIOSIS search terms and strategy.

Supplementary Figure 1: Summary of risk of bias questions with answers. “Yes” responses to questions (green) indicates low risk of bias; “No” responses to questions (red) indicates high risk of bias, using the tool described by Hoy et al. (2012).

Supplementary Figure 2: Summary of overall risk of bias for each included study, using the tool described by Hoy et al. (2012). Red indicates high risk of bias (two or more questions answered with “No”), orange indicates moderate risk of bias (only one question answered with “No”), green indicates low risk of bias (no questions answered with “No”).

Supplementary Figure 3: Five most common mutated genes in ever vs never smokers (A) decreasing size order for ever smokers (B) decreasing size order for never smokers. Light blue = ever smokers, dark blue = never smokers.

Supplementary Figure 4: Distinct missense mutations found in mutated *TP53* gene in NSCLC BM cohort.

Supplementary Figure 5: Distinct missense mutations found in mutated *EGFR* gene in NSCLC BM cohort.

Supplementary Figure 6: Distinct missense mutations found in mutated *KRAS* gene in NSCLC BM cohort.

Supplementary Figure 7: Distinct missense mutations found in mutated *CDKN2A* gene in NSCLC BM cohort.

Supplementary Figure 8: Distinct missense mutations found in mutated *STK11* gene in NSCLC BM cohort.

Supplementary Figure 9: Distinct missense mutations found in mutated *PIK3CA* gene in NSCLC BM cohort.

Supplementary Figure 10: Distinct missense mutations found in mutated *MYC* gene in NSCLC BM cohort.

Supplementary Figure 11: Distinct missense mutations found in mutated *TP53* gene in ever vs never smokers in NSCLC BM cohort.

Supplementary Figure 12: Distinct missense mutations found in mutated *EGFR* gene in ever vs never smokers in NSCLC BM cohort.

| Druggable Gene Category | Matching Gene Count | Matching Gene(s) | Non-Matching Gene(s) |
| --- | --- | --- | --- |
| [Clinically Actionable](https://www.dgidb.org/druggable_gene_categories/CLINICALLY%20ACTIONABLE?sources%5B%5D=BaderLabGenes&sources%5B%5D=CIViC&sources%5B%5D=COSMIC&sources%5B%5D=CarisMolecularIntelligence&sources%5B%5D=FoundationOneGenes&sources%5B%5D=GO&sources%5B%5D=GuideToPharmacology&sources%5B%5D=HingoraniCasas&sources%5B%5D=HopkinsGroom&sources%5B%5D=HumanProteinAtlas&sources%5B%5D=IDG&sources%5B%5D=MskImpact&sources%5B%5D=Oncomine&sources%5B%5D=Pharos&sources%5B%5D=RussLampel&sources%5B%5D=Tempus&sources%5B%5D=dGene) | 22 | EGFR, TP53, KRAS, CDKN2A, STK11, PIK3CA, MYC, CDKN2B, KEAP1, NKX2-1, RB1, MET, TERT, ERBB2, CTNNB1, LRP1B, MDM2, SMARCA4, ALK, PTEN, KMT2C, MCL1 |  |
| [Drug Resistance](https://www.dgidb.org/druggable_gene_categories/DRUG%20RESISTANCE?sources%5B%5D=BaderLabGenes&sources%5B%5D=CIViC&sources%5B%5D=COSMIC&sources%5B%5D=CarisMolecularIntelligence&sources%5B%5D=FoundationOneGenes&sources%5B%5D=GO&sources%5B%5D=GuideToPharmacology&sources%5B%5D=HingoraniCasas&sources%5B%5D=HopkinsGroom&sources%5B%5D=HumanProteinAtlas&sources%5B%5D=IDG&sources%5B%5D=MskImpact&sources%5B%5D=Oncomine&sources%5B%5D=Pharos&sources%5B%5D=RussLampel&sources%5B%5D=Tempus&sources%5B%5D=dGene) | 15 | EGFR, TP53, KRAS, CDKN2A, STK11, PIK3CA, RB1, MET, TERT, ERBB2, CTNNB1, LRP1B, ALK, PTEN, KMT2C | MYC, CDKN2B, KEAP1, NKX2-1, MDM2, SMARCA4, MCL1 |
| [Druggable Genome](https://www.dgidb.org/druggable_gene_categories/DRUGGABLE%20GENOME?sources%5B%5D=BaderLabGenes&sources%5B%5D=CIViC&sources%5B%5D=COSMIC&sources%5B%5D=CarisMolecularIntelligence&sources%5B%5D=FoundationOneGenes&sources%5B%5D=GO&sources%5B%5D=GuideToPharmacology&sources%5B%5D=HingoraniCasas&sources%5B%5D=HopkinsGroom&sources%5B%5D=HumanProteinAtlas&sources%5B%5D=IDG&sources%5B%5D=MskImpact&sources%5B%5D=Oncomine&sources%5B%5D=Pharos&sources%5B%5D=RussLampel&sources%5B%5D=Tempus&sources%5B%5D=dGene) | 13 | EGFR, TP53, STK11, PIK3CA, KEAP1, RB1, MET, TERT, ERBB2, CTNNB1, MDM2, ALK, MCL1 | KRAS, CDKN2A, MYC, CDKN2B, NKX2-1, LRP1B, SMARCA4, PTEN, KMT2C |
| [Kinase](https://www.dgidb.org/druggable_gene_categories/KINASE?sources%5B%5D=BaderLabGenes&sources%5B%5D=CIViC&sources%5B%5D=COSMIC&sources%5B%5D=CarisMolecularIntelligence&sources%5B%5D=FoundationOneGenes&sources%5B%5D=GO&sources%5B%5D=GuideToPharmacology&sources%5B%5D=HingoraniCasas&sources%5B%5D=HopkinsGroom&sources%5B%5D=HumanProteinAtlas&sources%5B%5D=IDG&sources%5B%5D=MskImpact&sources%5B%5D=Oncomine&sources%5B%5D=Pharos&sources%5B%5D=RussLampel&sources%5B%5D=Tempus&sources%5B%5D=dGene) | 11 | EGFR, TP53, CDKN2A, STK11, PIK3CA, CDKN2B, RB1, MET, ERBB2, ALK, PTEN | KRAS, MYC, KEAP1, NKX2-1, TERT, CTNNB1, LRP1B, MDM2, SMARCA4, MCL1, KMT2C |
| [Enzyme](https://www.dgidb.org/druggable_gene_categories/ENZYME?sources%5B%5D=BaderLabGenes&sources%5B%5D=CIViC&sources%5B%5D=COSMIC&sources%5B%5D=CarisMolecularIntelligence&sources%5B%5D=FoundationOneGenes&sources%5B%5D=GO&sources%5B%5D=GuideToPharmacology&sources%5B%5D=HingoraniCasas&sources%5B%5D=HopkinsGroom&sources%5B%5D=HumanProteinAtlas&sources%5B%5D=IDG&sources%5B%5D=MskImpact&sources%5B%5D=Oncomine&sources%5B%5D=Pharos&sources%5B%5D=RussLampel&sources%5B%5D=Tempus&sources%5B%5D=dGene) | 6 | STK11, KEAP1, TERT, MDM2, PTEN, KMT2C | EGFR, TP53, KRAS, CDKN2A, PIK3CA, MYC, CDKN2B, NKX2-1, RB1, MET, ERBB2, CTNNB1, LRP1B, SMARCA4, ALK, MCL1 |
| [Tumor Suppressor](https://www.dgidb.org/druggable_gene_categories/TUMOR%20SUPPRESSOR?sources%5B%5D=BaderLabGenes&sources%5B%5D=CIViC&sources%5B%5D=COSMIC&sources%5B%5D=CarisMolecularIntelligence&sources%5B%5D=FoundationOneGenes&sources%5B%5D=GO&sources%5B%5D=GuideToPharmacology&sources%5B%5D=HingoraniCasas&sources%5B%5D=HopkinsGroom&sources%5B%5D=HumanProteinAtlas&sources%5B%5D=IDG&sources%5B%5D=MskImpact&sources%5B%5D=Oncomine&sources%5B%5D=Pharos&sources%5B%5D=RussLampel&sources%5B%5D=Tempus&sources%5B%5D=dGene) | 5 | TP53, CDKN2A, STK11, RB1, MDM2 | EGFR, KRAS, PIK3CA, MYC, CDKN2B, KEAP1, NKX2-1, MET, TERT, ERBB2, CTNNB1, LRP1B, SMARCA4, ALK, PTEN, MCL1, KMT2C |
| [Transcription Factor](https://www.dgidb.org/druggable_gene_categories/TRANSCRIPTION%20FACTOR?sources%5B%5D=BaderLabGenes&sources%5B%5D=CIViC&sources%5B%5D=COSMIC&sources%5B%5D=CarisMolecularIntelligence&sources%5B%5D=FoundationOneGenes&sources%5B%5D=GO&sources%5B%5D=GuideToPharmacology&sources%5B%5D=HingoraniCasas&sources%5B%5D=HopkinsGroom&sources%5B%5D=HumanProteinAtlas&sources%5B%5D=IDG&sources%5B%5D=MskImpact&sources%5B%5D=Oncomine&sources%5B%5D=Pharos&sources%5B%5D=RussLampel&sources%5B%5D=Tempus&sources%5B%5D=dGene) | 4 | MYC, NKX2-1, RB1, CTNNB1 | EGFR, TP53, KRAS, CDKN2A, STK11, PIK3CA, CDKN2B, KEAP1, MET, TERT, ERBB2, LRP1B, MDM2, SMARCA4, ALK, PTEN, MCL1, KMT2C |
| [Transcription Factor Complex](https://www.dgidb.org/druggable_gene_categories/TRANSCRIPTION%20FACTOR%20COMPLEX?sources%5B%5D=BaderLabGenes&sources%5B%5D=CIViC&sources%5B%5D=COSMIC&sources%5B%5D=CarisMolecularIntelligence&sources%5B%5D=FoundationOneGenes&sources%5B%5D=GO&sources%5B%5D=GuideToPharmacology&sources%5B%5D=HingoraniCasas&sources%5B%5D=HopkinsGroom&sources%5B%5D=HumanProteinAtlas&sources%5B%5D=IDG&sources%5B%5D=MskImpact&sources%5B%5D=Oncomine&sources%5B%5D=Pharos&sources%5B%5D=RussLampel&sources%5B%5D=Tempus&sources%5B%5D=dGene) | 4 | TP53, NKX2-1, RB1, CTNNB1 | EGFR, KRAS, CDKN2A, STK11, PIK3CA, MYC, CDKN2B, KEAP1, MET, TERT, ERBB2, LRP1B, MDM2, SMARCA4, ALK, PTEN, MCL1, KMT2C |
| [Tyrosine Kinase](https://www.dgidb.org/druggable_gene_categories/TYROSINE%20KINASE?sources%5B%5D=BaderLabGenes&sources%5B%5D=CIViC&sources%5B%5D=COSMIC&sources%5B%5D=CarisMolecularIntelligence&sources%5B%5D=FoundationOneGenes&sources%5B%5D=GO&sources%5B%5D=GuideToPharmacology&sources%5B%5D=HingoraniCasas&sources%5B%5D=HopkinsGroom&sources%5B%5D=HumanProteinAtlas&sources%5B%5D=IDG&sources%5B%5D=MskImpact&sources%5B%5D=Oncomine&sources%5B%5D=Pharos&sources%5B%5D=RussLampel&sources%5B%5D=Tempus&sources%5B%5D=dGene) | 4 | EGFR, MET, ERBB2, ALK | TP53, KRAS, CDKN2A, STK11, PIK3CA, MYC, CDKN2B, KEAP1, NKX2-1, RB1, TERT, CTNNB1, LRP1B, MDM2, SMARCA4, PTEN, MCL1, KMT2C |
| [Cell Surface](https://www.dgidb.org/druggable_gene_categories/CELL%20SURFACE?sources%5B%5D=BaderLabGenes&sources%5B%5D=CIViC&sources%5B%5D=COSMIC&sources%5B%5D=CarisMolecularIntelligence&sources%5B%5D=FoundationOneGenes&sources%5B%5D=GO&sources%5B%5D=GuideToPharmacology&sources%5B%5D=HingoraniCasas&sources%5B%5D=HopkinsGroom&sources%5B%5D=HumanProteinAtlas&sources%5B%5D=IDG&sources%5B%5D=MskImpact&sources%5B%5D=Oncomine&sources%5B%5D=Pharos&sources%5B%5D=RussLampel&sources%5B%5D=Tempus&sources%5B%5D=dGene) | 2 | EGFR, MET | TP53, KRAS, CDKN2A, STK11, PIK3CA, MYC, CDKN2B, KEAP1, NKX2-1, RB1, TERT, ERBB2, CTNNB1, LRP1B, MDM2, SMARCA4, ALK, PTEN, MCL1, KMT2C |
| [Serine Threonine Kinase](https://www.dgidb.org/druggable_gene_categories/SERINE%20THREONINE%20KINASE?sources%5B%5D=BaderLabGenes&sources%5B%5D=CIViC&sources%5B%5D=COSMIC&sources%5B%5D=CarisMolecularIntelligence&sources%5B%5D=FoundationOneGenes&sources%5B%5D=GO&sources%5B%5D=GuideToPharmacology&sources%5B%5D=HingoraniCasas&sources%5B%5D=HopkinsGroom&sources%5B%5D=HumanProteinAtlas&sources%5B%5D=IDG&sources%5B%5D=MskImpact&sources%5B%5D=Oncomine&sources%5B%5D=Pharos&sources%5B%5D=RussLampel&sources%5B%5D=Tempus&sources%5B%5D=dGene) | 2 | STK11, PIK3CA | EGFR, TP53, KRAS, CDKN2A, MYC, CDKN2B, KEAP1, NKX2-1, RB1, MET, TERT, ERBB2, CTNNB1, LRP1B, MDM2, SMARCA4, ALK, PTEN, MCL1, KMT2C |
| [Phosphatidylinositol 3 Kinase](https://www.dgidb.org/druggable_gene_categories/PHOSPHATIDYLINOSITOL%203%20KINASE?sources%5B%5D=BaderLabGenes&sources%5B%5D=CIViC&sources%5B%5D=COSMIC&sources%5B%5D=CarisMolecularIntelligence&sources%5B%5D=FoundationOneGenes&sources%5B%5D=GO&sources%5B%5D=GuideToPharmacology&sources%5B%5D=HingoraniCasas&sources%5B%5D=HopkinsGroom&sources%5B%5D=HumanProteinAtlas&sources%5B%5D=IDG&sources%5B%5D=MskImpact&sources%5B%5D=Oncomine&sources%5B%5D=Pharos&sources%5B%5D=RussLampel&sources%5B%5D=Tempus&sources%5B%5D=dGene) | 2 | KEAP1, SMARCA4 | EGFR, TP53, KRAS, CDKN2A, STK11, PIK3CA, MYC, CDKN2B, NKX2-1, RB1, MET, TERT, ERBB2, CTNNB1, LRP1B, MDM2, ALK, PTEN, MCL1, KMT2C |
| [Protein Phosphatase](https://www.dgidb.org/druggable_gene_categories/PROTEIN%20PHOSPHATASE?sources%5B%5D=BaderLabGenes&sources%5B%5D=CIViC&sources%5B%5D=COSMIC&sources%5B%5D=CarisMolecularIntelligence&sources%5B%5D=FoundationOneGenes&sources%5B%5D=GO&sources%5B%5D=GuideToPharmacology&sources%5B%5D=HingoraniCasas&sources%5B%5D=HopkinsGroom&sources%5B%5D=HumanProteinAtlas&sources%5B%5D=IDG&sources%5B%5D=MskImpact&sources%5B%5D=Oncomine&sources%5B%5D=Pharos&sources%5B%5D=RussLampel&sources%5B%5D=Tempus&sources%5B%5D=dGene) | 1 | [PIK3CA](https://www.dgidb.org/genes/PIK3CA) | EGFR, TP53, KRAS, CDKN2A, STK11, MYC, CDKN2B, KEAP1, NKX2-1, RB1, MET, TERT, ERBB2, CTNNB1, LRP1B, MDM2, SMARCA4, ALK, PTEN, MCL1, KMT2C |
| [Pten Family](https://www.dgidb.org/druggable_gene_categories/PTEN%20FAMILY?sources%5B%5D=BaderLabGenes&sources%5B%5D=CIViC&sources%5B%5D=COSMIC&sources%5B%5D=CarisMolecularIntelligence&sources%5B%5D=FoundationOneGenes&sources%5B%5D=GO&sources%5B%5D=GuideToPharmacology&sources%5B%5D=HingoraniCasas&sources%5B%5D=HopkinsGroom&sources%5B%5D=HumanProteinAtlas&sources%5B%5D=IDG&sources%5B%5D=MskImpact&sources%5B%5D=Oncomine&sources%5B%5D=Pharos&sources%5B%5D=RussLampel&sources%5B%5D=Tempus&sources%5B%5D=dGene) | 1 | [PTEN](https://www.dgidb.org/genes/PTEN) | EGFR, TP53, KRAS, CDKN2A, STK11, PIK3CA, MYC, CDKN2B, KEAP1, NKX2-1, RB1, MET, TERT, ERBB2, CTNNB1, LRP1B, MDM2, SMARCA4, ALK, MCL1, KMT2C |
| [Rna Directed Dna Polymerase](https://www.dgidb.org/druggable_gene_categories/RNA%20DIRECTED%20DNA%20POLYMERASE?sources%5B%5D=BaderLabGenes&sources%5B%5D=CIViC&sources%5B%5D=COSMIC&sources%5B%5D=CarisMolecularIntelligence&sources%5B%5D=FoundationOneGenes&sources%5B%5D=GO&sources%5B%5D=GuideToPharmacology&sources%5B%5D=HingoraniCasas&sources%5B%5D=HopkinsGroom&sources%5B%5D=HumanProteinAtlas&sources%5B%5D=IDG&sources%5B%5D=MskImpact&sources%5B%5D=Oncomine&sources%5B%5D=Pharos&sources%5B%5D=RussLampel&sources%5B%5D=Tempus&sources%5B%5D=dGene) | 1 | [PTEN](https://www.dgidb.org/genes/PTEN) | EGFR, TP53, KRAS, CDKN2A, STK11, PIK3CA, MYC, CDKN2B, KEAP1, NKX2-1, RB1, MET, TERT, ERBB2, CTNNB1, LRP1B, MDM2, SMARCA4, ALK, MCL1, KMT2C |
| [Transcription Factor Binding](https://www.dgidb.org/druggable_gene_categories/TRANSCRIPTION%20FACTOR%20BINDING?sources%5B%5D=BaderLabGenes&sources%5B%5D=CIViC&sources%5B%5D=COSMIC&sources%5B%5D=CarisMolecularIntelligence&sources%5B%5D=FoundationOneGenes&sources%5B%5D=GO&sources%5B%5D=GuideToPharmacology&sources%5B%5D=HingoraniCasas&sources%5B%5D=HopkinsGroom&sources%5B%5D=HumanProteinAtlas&sources%5B%5D=IDG&sources%5B%5D=MskImpact&sources%5B%5D=Oncomine&sources%5B%5D=Pharos&sources%5B%5D=RussLampel&sources%5B%5D=Tempus&sources%5B%5D=dGene) | 1 | [TERT](https://www.dgidb.org/genes/TERT) | EGFR, TP53, KRAS, CDKN2A, STK11, PIK3CA, MYC, CDKN2B, KEAP1, NKX2-1, RB1, MET, ERBB2, CTNNB1, LRP1B, MDM2, SMARCA4, ALK, PTEN, MCL1, KMT2C |
| [Transporter](https://www.dgidb.org/druggable_gene_categories/TRANSPORTER?sources%5B%5D=BaderLabGenes&sources%5B%5D=CIViC&sources%5B%5D=COSMIC&sources%5B%5D=CarisMolecularIntelligence&sources%5B%5D=FoundationOneGenes&sources%5B%5D=GO&sources%5B%5D=GuideToPharmacology&sources%5B%5D=HingoraniCasas&sources%5B%5D=HopkinsGroom&sources%5B%5D=HumanProteinAtlas&sources%5B%5D=IDG&sources%5B%5D=MskImpact&sources%5B%5D=Oncomine&sources%5B%5D=Pharos&sources%5B%5D=RussLampel&sources%5B%5D=Tempus&sources%5B%5D=dGene) | 1 | TP53 | EGFR, KRAS, CDKN2A, STK11, PIK3CA, MYC, CDKN2B, KEAP1, NKX2-1, RB1, MET, TERT, ERBB2, CTNNB1, LRP1B, MDM2, SMARCA4, ALK, PTEN, MCL1, KMT2C |
| Methyl Transferase | 1 | KMT2C | EGFR, TP53, KRAS, CDKN2A, STK11, PIK3CA, MYC, CDKN2B, KEAP1, NKX2-1, RB1, MET, TERT, ERBB2, CTNNB1, LRP1B, MDM2, SMARCA4, ALK, PTEN, MCL1 |
| NUCLEAR HORMONE RECEPTOR | 1 | [KMT2C](https://www.dgidb.org/genes/TP53) | EGFR, TP53, KRAS, CDKN2A, STK11, PIK3CA, MYC, CDKN2B, KEAP1, NKX2-1, RB1, MET, TERT, ERBB2, CTNNB1, LRP1B, MDM2, SMARCA4, ALK, PTEN, MCL1 |

Supplementary Table 5: DGIdb generated druggable categories for top mutated genes in the NSCLC BM cohort.

| Altered gene/pathway | Interventions | Phases | Title | NCT Number |
| --- | --- | --- | --- | --- |
| EGFR T790M | Drug: AZD9291 | Phase 2 | Study of AZD9291 in NSCLC Patients Harboring T790M Mutation Who Failed EGFR TKI and With Brain and/or LMS | NCT03257124 |
| EGFR exon 20 insertion | Drug: furmonertinib 240 mg\|Drug: furmonertinib 160 mg\|Drug: platinum-based chemotherapy | Phase 3 | Study to Compare Furmonertinib to Platinum-Based Chemotherapy for Patients With Locally Advanced or Metastatic Non-Small Cell Lung Cancer | NCT05607550 |
| EGFR | Drug: Tesevatinib | Phase 2 | Phase 2 Study of Study of Tesevatinib in Subjects With NSCLC and Brain or Leptomeningeal Metastases | NCT02616393 |
| EGFR | Radiation: Stereotactic surgery | Not Applicable | A Study to Evaluate the Efficacy of Osimertinib With Early Intervention SRS Treatment Compared to the Continuation of Osimertinib Alone, in Patients With EGFR Mutated NSCLC and Asymptomatic Brain Metastases | NCT05033691 |
| EGFR | Drug: Dacomitinib | Phase 2 | Central Nervous System(CNS) Efficacy of Dacomitinib | NCT04675008 |
| EGFR | Drug: Almonertinib\|Radiation: Stereotactic Radiotherapy(SRT) or Stereotactic Radiosurgery(SRS) or Whole-Brain Radiotherapy(WBRT) | Phase 2 | Almonertinib Combined With Cerebral Radiation Treat Brain Metastases From EGFR Positive NSCLC | NCT04905550 |
| EGFR | Drug: lazertinib(YH25448) | Phase 2 | Lazertinib in Patients With NSCLC With Asymptomatic or Mild Symptomatic Brain Metastases After Failure of EGFR TKI. | NCT05326425 |
| EGFR | Drug: Amivantamab\|Drug: Lazertinib | Phase 2 | A Study of Amivantamab and Lazertinib in People With Non-Small Cell Lung Cancer (NSCLC) | NCT04965090 |
| EGFR | Drug: AZD3759\|Drug: AZD9291 | Phase 1 | Oral Epidermal Growth Factor Receptor Tyrosine Kinase Inhibitors, AZD3759 or AZD9291, in Patients Who Have Advanced Non-Small Cell Lung Cancer | NCT02228369 |
| EGFR | Drug: AZD3759\|Drug: Erlotinib\|Drug: Gefitinib | Phase 2\|Phase 3 | First Line Treatment in EGFR Mutation Positive Advanced NSCLC Patients With Central Nervous System (CNS) Metastases | NCT03653546 |
| EGFR | Biological: Bevacizumab\|Other: Laboratory Biomarker Analysis\|Drug: Osimertinib | Phase 2 | Osimertinib With or Without Bevacizumab in Treating Patients With EGFR Positive Non-small Cell Lung Cancer and Brain Metastases | NCT02971501 |
| EGFR | Drug: icotinib\|Radiation: Whole brain radiotherapy | Phase 1\|Phase 2 | Icotinib Combined With WBRT For NSCLC Patients With Brain Metastases and EGFR Mutation | NCT01516983 |
| EGFR | Drug: Anlotinib | Phase 2 | the Efficacy and Safety of Anlotinib Combined With Almonertinib in the First-line Treatment of Patients With Brain Metastases From EGFR Mutation-positive Non-small Cell Lung Cancer | NCT04978753 |
| EGFR | Drug: Osimertinib\|Radiation: Stereotactic radiotherapy | Phase 2 | Study of Osimertinib + SRS vs Osimertinib Alone for Brain Metastases in EGFR Positive Patients With NSCLC | NCT03769103 |
| EGFR | Drug: osimertinib oral and bevazizumab intravenously | Phase 3 | Osimertinib Combined With Bevacizumab in Patients With Brain Metastasis Epidermal Growth Factor Receptor (EGFR) Mutation Positive Metastatic Non-Small Cell Lung Cancer | NCT05104281 |
| EGFR | Drug: Osimertinib\|Drug: [11C]osimertinib | Phase 1 | Open-label PET Study With [11C]Osimertinib in Patients With EGFRm NSCLC and Brain Metastases | NCT03463525 |
| EGFR | Drug: Osimertinib | Phase 1 | Osimertinib With Stereotactic Radiosurgery (SRS) in Brain Metastases From EGFR Positive NSCLC | NCT03535363 |
| EGFR | Drug: Osimertinib | Phase 2 | Study of Osimertinib in Patients With a Lung Cancer With Brain or Leptomeningeal Metastases With EGFR Mutation | NCT04233021 |
| EGFR | Drug: Gefitinib and Pemetrexed/platinum\|Drug: Gefitinib mono-therapy | Phase 3 | Gefitinib With or Without Chemotherapy in Brain Metastases From Non-small Cell Lung Cancer | NCT01951469 |
| EGFR | Drug: Keynatinib | Phase 2 | Keynatinib in Treated Patients With NSCLC and Brain Metastases | NCT04824079 |
| EGFR | Drug: Osimertinib (AZD9291) pemetrexed cisplatin or carboplatin\|Drug: Placebo for osimertinib (AZD9291) pemetrexed cisplatin or carboplatin | Phase 3 | A Study to Evaluate Chemotherapy Plus Osimertinib Against Chemotherapy Plus Placebo in Patients With Non-small Cell Lung Cancer (NSCLC) | NCT04765059 |
| EGFR | Drug: Osimertinib\|Radiation: Stereotactic Radiosurgery (SRS) | Phase 2 | A Randomised Phase II Trial of Osimertinib With or Without SRS for EGFR Mutated NSCLC With Brain Metastases | NCT03497767 |
| EGFR | Radiation: whole brain radiation(WBI)\|Drug: Icotinib | Phase 3 | Icotinib or Whole Brain Irradiation in EGFR-mutant Lung Cancer | NCT01724801 |
| EGFR | Drug: almonertinib\|Drug: LM-first line treatment\|Drug: LM-second line treatment | Not Applicable | A Dose Exploration Study of Almonertinib for EGFRm NSCLC Patients With Brain/Leptomeningeal Metastasis (ARTISTRY) | NCT04778800 |
| EGFR | Drug: TY-9591 Tablets | Phase 2 | Study of TY-9591 in Patients With a Lung Cancer With Brain or Leptomeningeal Metastases With EGFR Mutation | NCT05146219 |
| EGFR | Biological: Bevacizumab\|Drug: Osimertinib | Phase 3 | Osimertinib With or Without Bevacizumab as Initial Treatment for Patients With EGFR-Mutant Lung Cancer | NCT04181060 |
| KRAS G12C | Drug: AMG 510\|Drug: MVASI | Phase 1\|Phase 2 | A Phase I/II Study of AMG 510 in Combination With MVASI in Patients With Advanced, Unresectable or Metastatic KRAS G12C Mutant NSCLC With Asymptomatic Brain Metastasis | NCT05180422 |
| MET | Drug: Capmatinib |  | Real-World Assessment of Clinical Outcomes in Metastatic NSCLC Patients With MET Exon 14 Skipping Mutation and Brain Metastases Treated With Capmatinib | NCT05675683 |
| ALK | Drug: Brigatinib | Phase 2 | A Study to Evaluate the Efficacy of Brigatinib (AP26113) in Participants With Anaplastic Lymphoma Kinase (ALK)-Positive, Non-small Cell Lung Cancer (NSCLC) Previously Treated With Crizotinib | NCT02094573 |
| ALK | Drug: TPX-0131 | Phase 1\|Phase 2 | A Study of TPX-0131, a Novel Oral ALK Tyrosine Kinase Inhibitor, in Patients With ALK+ Advanced or Metastatic NSCLC | NCT04849273 |
| ALK | Drug: Alectinib\|Drug: Bevacizumab | Phase 2 | Alectinib in Combination With Bevacizumab in ALK Positive NSCLC | NCT03779191 |
| RET | Drug: Selpercatinib | Phase 2 | Targeted Treatment for RET Fusion-Positive Advanced Non-Small Cell Lung Cancer (A LUNG-MAP Treatment Trial) | NCT04268550 |
| RET | Drug: Selpercatinib\|Drug: Carboplatin\|Drug: Cisplatin\|Drug: Pemetrexed\|Drug: Pembrolizumab | Phase 3 | A Study of Selpercatinib (LY3527723) in Participants With Advanced or Metastatic RET Fusion-Positive Non-Small Cell Lung Cancer | NCT04194944 |
| ROS1 | Drug: Lorlatinib | Phase 2 | Lorlatinib After Failure of First-line TKI in Patients With Advanced ROS1-positive NSCLC (ALBATROS) | NCT04621188 |
| Her2 | Drug: BI 1810631 | Phase 1 | A Study to Test Different Doses of BI 1810631 in People With Different Types of Advanced Cancer (Solid Tumours With Changes in the HER2 Gene) | NCT04886804 |
| EGFR/ALK | Radiation: Stereotactic radiosurgery/whole brain radiotherapy\|Drug: Tyrosine kinase inhibitor | Phase 3 | Observation or Upfront Cranial RT in Oncogene Mutated NSCLC With Asymptomatic BM: A Phase III RCT | NCT05236946 |
| EGFR/ALK/ROS1 | Radiation: Stereotactic radiosurgery\|Drug: Immune checkpoint inhibitor | Phase 3 | Immunotherapy or Targeted Therapy With or Without Stereotactic Radiosurgery for Patients With Brain Metastases From Melanoma or Non-small Cell Lung Cancer | NCT05522660 |
| ALK/ROS1 | Drug: PF-06463922\|Drug: Crizotinib | Phase 1\|Phase 2 | A Study Of PF-06463922 An ALK/ROS1 Inhibitor In Patients With Advanced Non Small Cell Lung Cancer With Specific Molecular Alterations | NCT01970865 |

Supplementary Table 6: Clinical trials generated from clinicaltrials.gov search for drugs targeting the top mutated genes in NSCLC BM.
